# 罕见*EGFR* L833V/H835L复合突变的肺腺癌患者1例及文献回顾

**DOI:** 10.3779/j.issn.1009-3419.2023.102.36

**Published:** 2023-10-20

**Authors:** Yongen MIAO, Yukun WANG, Ping LI, Min TAN, Tingting WEN, Changhui WANG, Shuanshuan XIE

**Affiliations:** ^1^200072 上海，同济大学附属第十人民医院呼吸与危重症医学科; ^1^Department of Respiratory and Critical Care Medicine, Shanghai Tenth People’s Hospital, Tongji University, Shanghai 200072, China; ^2^200092 上海，同济大学医学院; ^2^Tongji University School of Medicine, Shanghai 200092, China

**Keywords:** 肺肿瘤, 表皮生长因子受体, 复合突变, L833V/H835L, 阿法替尼, Lung neoplasms, Epidermal growth factor receptor, Compound variants, L833V/H835L, Afatinib

## Abstract

表皮生长因子受体（epidermal growth factor receptor, EGFR）突变是非小细胞肺癌（non-small cell lung cancer, NSCLC）发生发展过程中最常见的驱动基因，其中18-21号外显子的突变常见，尤其是19号外显子的缺失和21号外显子L858R点突变最为常见，但是EGFR L833V/H835L罕见基因复合突变的发生率非常低，患者数量很少，相关临床数据和治疗方法的证据也不足。部分EGFR-酪氨酸激酶抑制剂（EGFR-tyrosine kinase inhibitors, EGFR-TKIs）在治疗伴有罕见基因突变的肺癌患者方面同样具有良好疗效。本文报道了1例携带EGFR L833V/H835L罕见基因复合突变的NSCLC患者，在给予阿法替尼治疗5个月后，计算机断层扫描（computed tomography, CT）显示肺部病灶缩小，患者对阿法替尼联合安罗替尼治疗反应良好。同时，我们还对以往报道的EGFR L833V/H835L罕见基因复合突变的NSCLC患者进行了整理，总结了该类患者的特点及应用不同种类EGFR-TKIs治疗的效果。

## Abstract (en)

Epidermal growth factor receptor (EGFR) mutations are the most common driver genes in the development of non-small cell lung cancer (NSCLC), of which mutations in exons 18-21 are frequent, especially the loss of exon 19 and exon 21 L858R mutation are the most frequent. Other rare gene mutations are rare. Simultaneous occurrence of two or more rare EGFR mutations are extremely rare in lung cancer, and the incidence of EGFR L833V/H835L rare gene compound mutations is very low, and there is little clinical data and evidence of relevant treatment methods. Some EGFR-tyrosine kinase inhibitors (EGFR-TKIs) are effective in treating lung cancer patients with rare gene mutations. In this article, we reported a case of NSCLC patient with a rare gene compound mutation EGFR L833V/H835L, who responded to Afatinib in combination with Anilotinib treatment well after 5 months of treatment, and computed tomography (CT) showed shrinkage of lung lesions. Meanwhile, we also compiled previously reported NSCLC patients with EGFR L833V/H835L rare gene compound mutation and summarized the characteristics of this group of patients and the effect of applying different kinds of EGFR-TKIs treatment.

肺癌是目前人类最常见的恶性肿瘤之一，其发病率和死亡率居首位。表皮生长因子受体（epidermal growth factor receptor, EGFR）突变是非小细胞肺癌（non-small cell lung cancer, NSCLC）患者最常见的驱动基因，10%-15%的高加索NSCLC患者和50%以上的亚洲NSCLC患者存在EGFR基因突变^[1⇓-3]^，其中18-21号外显子的突变常见，尤其是19号外显子的缺失和21号外显子L858R点突变最为常见^[4]^。罕见EGFR变异指的是一组发生率较低的EGFR基因突变，相对罕见变异包括G719X、L861Q和S768I，极罕见变异包括L833V/H835L和其他复合变异^[5]^。目前，随着EGFR-酪氨酸激酶抑制剂（EGFR-tyrosine kinase inhibitors, EGFR-TKIs）在NSCLC患者中的应用，EGFR基因突变肺癌患者的预后得到明显改善。但是在临床上EGFR-TKIs主要治疗EGFR突变患者，关于治疗罕见突变的信息较少。本文报道了临床中发现的1例EGFR 21号外显子L833V/H835L罕见基因复合突变的肺癌患者，该患者在接受阿法替尼治疗后，治疗效果良好。

## 1 病例资料

### 1.1 患者资料

患者，女性，40岁，因“左侧胸痛4天”于2018年3月12日入院。初次入院全身评估：体格检查：无明显阳性体征；肺部计算机断层扫描（computed tomography, CT）（图1）提示：左肺上叶尖后段见约2.5 cm×1.5 cm×4.0 cm的软组织密度影。于2018年3月14日行胸腔镜下肺叶切除术+纵隔淋巴结清扫术，术后病理结果：胸膜下一肿块，切面呈灰白色，质中，与周围组织分界不清；（左肺上叶）浸润性肺腺癌，腺泡为主型（腺泡约40%，实体约30%，贴壁约20%，微乳头约10%），肿块大小2.7 cm×2.2 cm×2 cm累及支气管管壁，可见气腔播散，可疑脉管内癌栓，未见胸膜累及，支气管切缘未见累及，未见癌转移，肿瘤原发灶-淋巴结-转移（tumor-node-metastasis, TNM）分期为IB期。术后行基因检测，结果未见异常突变。既往有高血压病史6年，最高180/90 mmHg，平素服用“马来酸左旋氨氯地平2.5 mg，qd”治疗，血压控制可。未发现其他疾病。患者于1998年行阑尾切除术，2006年行剖宫产手术，否认其他重大手术外伤史。否认糖尿病、慢性阻塞性肺疾病（chronic obstructive pulmonary disease, COPD）等慢性疾病史，否认药物及食物过敏史。术后进行化疗并在随访治疗过程中定期复查肿瘤标志物及肺部CT并据此调整化疗方案（图2）。期间正电子发射计算机断层扫描（positron emission tomography/CT, PET/CT）检查发现右侧肱骨头骨质破坏，肱骨上段不均匀密度增高伴氟代脱氧葡萄糖（flurodeoxyglucose, FDG）异常代谢增高，骨转移。后因疾病进展（progressive disease, PD），癌胚抗原（carcinoembryonic antigen, CEA）持续升高，于2023年5月9日再次进行基因检测。通过肺穿刺取得患者肺组织，由广州迈景基因医学检验实验室负责检验，采用多重聚合酶链式反应（polymerase chain reaction, PCR）技术建库和第二代测序技术（next generation sequencing, NGS）测序，结果显示：肿瘤病理诊断为腺癌，肿瘤类型为NSCLC，样本肿瘤细胞比例为45%，EGFR基因21号外显子L833V错义突变，丰度为6.5%；EGFR基因21号外显子H835L错义突变，丰度为6.5%，未发现其他具有临床意义的突变。血常规：红细胞5.36×10^12^/L，白细胞7.81×10^9^/L，血小板270×10^9^/L。凝血功能：纤维蛋白原4.83 g/L，D-二聚体0.30 mg/L，国际标准化比值（international normalized ratio, INR）1.01。血清肿瘤标志物：CEA 48.70 ng/mL，甲胎蛋白（alpha fetoprotein, AFP）3.08 ng/mL，糖类抗原153（cancer antigen 153, CA153）16.80 U/mL，糖类抗原199（cancer antigen 199, CA199）25.70 U/mL，铁蛋白436 ng/mL，神经元特异性烯醇化酶（neuron-specific enolase, NSE）21 ng/mL。肺部CT显示：左肺上叶切除术后改变，两肺多发结节灶，提示肺多发转移瘤；左肺下叶内前基底段少许慢性炎症改变；主动脉壁局部钙化；心包前壁轻度增厚，肺动脉增宽。

**图1:**
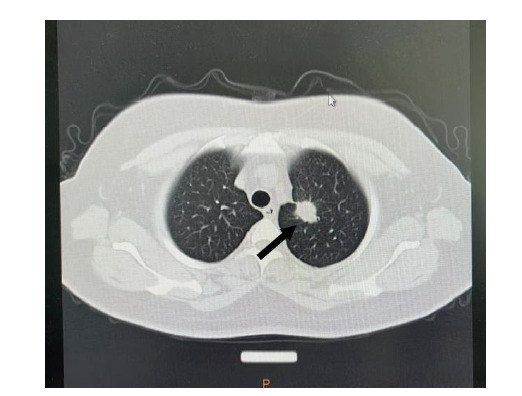
左肺上叶尖后段占位性病变（箭头所示）

**图2:**
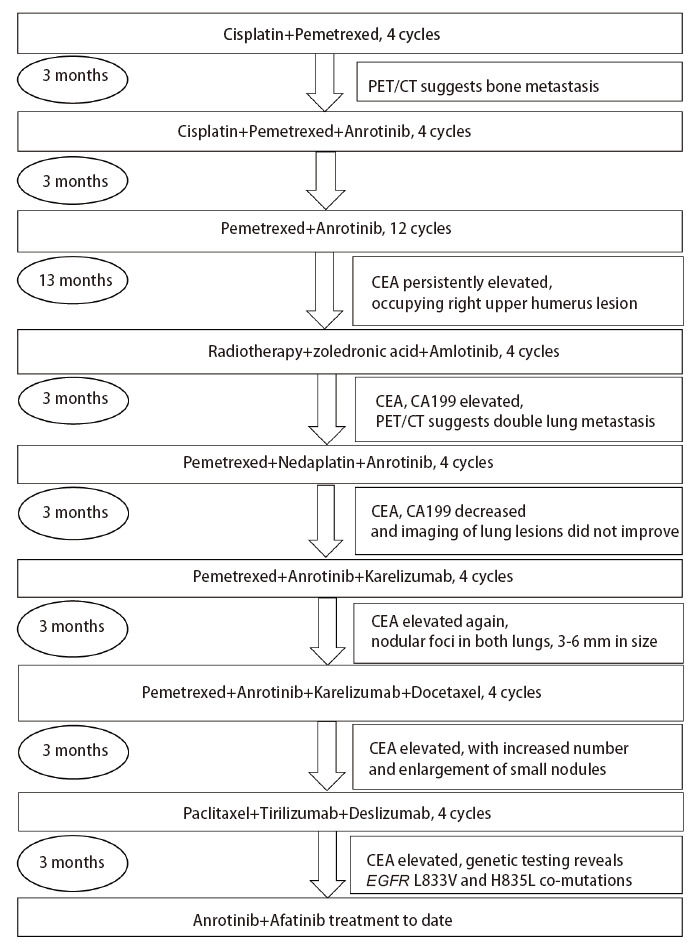
2018至2023年患者治疗流程图

### 1.2 诊断

2023年5月患者复查，结合患者CT、PET/CT表现、血清学检查及病理组织活检结果，考虑患者目前的诊断为：①支气管肺癌，原发性，左肺上叶，浸润性腺癌，T4N1M1B，IV期；②右侧肱骨上段骨继发性恶性肿瘤；③肺部感染。

### 1.3 治疗经过

2018年4月20日-2018年6月29日，术后予顺铂+培美曲赛进行化疗，共4个疗程。首次方案治疗的最佳疗效评估为疾病稳定（stable disease, SD）。

2019年5月17日-2020年9月2日，因复查结果显示：CEA升高达111.50 ng/mL，PET/CT示右侧肱骨头骨质破坏伴FDG异常代谢增高，考虑骨转移。化疗方案更改为顺铂+培美曲赛+安罗替尼，共4个疗程，随诊CT检查结果显示，肺部靶病灶最大径之和缩小，最佳疗效评估为SD。此后采用培美曲赛+安罗替尼维持治疗，共12个疗程，复查发现PD。

2020年10月20日-2021年1月5日，因CEA持续升高，PET/CT示右侧肱骨上端占位性病变，提示右侧肱骨转移。进行放射治疗[放疗靶区：肿瘤靶区（gross tumor volume, GTV）：右肱骨头可见转移灶；计划靶区（planning target volume, PTV）：GTV外放0.5 cm；放疗剂量：PTV 50 Gy/25 Fx]，放疗期间继续使用安罗替尼靶向治疗。2021年1月5日-2021年3月31日给予唑来膦酸抑制骨质破坏，共4个疗程。最佳疗效评估为SD。

2021年5月13日-2021年7月19日因CEA、CA199明显升高，PET/CT提示双肺转移，无特殊突变基因，给予培美曲赛+奈达铂+安罗替尼，共4个疗程。最佳疗效评估为SD。

2021年9月28日-2022年3月18日，因CEA、CA199明显下降，肺部病灶影像学无明显改善，给予培美曲赛+卡瑞利珠单抗+安罗替尼，共6个疗程。最佳疗效评估为SD。

2022年6月14日-2022年11月1日，因CEA持续升高，肺部CT结果显示：右肺及左肺下叶基底段粟粒、结节灶直径3-6 mm，提示病灶增大，给予培美曲赛+安罗替尼+卡瑞利珠单抗+多西他赛，共4个疗程。复查发现PD。

2022年11月1日-2023年3月29日，因肺部CT结果显示：双肺多发小结节，部分呈磨玻璃密度，部分病灶稍增大，提示PD，给予紫杉醇+替雷利珠单抗，共4个疗程。同时定期给予地舒单抗抑制骨质破坏。最佳疗效评估为SD。

2023年5月4日至今，因CEA持续升高，经肺穿刺取得患者肺组织，采用PCR技术建库和NGS，基因检测结果显示EGFR L833V/H835L复合突变，调整化疗方案为安罗替尼+阿法替尼。最佳疗效为SD（图2）。

### 1.4 治疗结果、随访及转归

经过安罗替尼+阿法替尼治疗后，患者胸痛等症状明显改善，CEA明显下降，肺部CT（图3）提示病灶吸收情况良好，最佳治疗效果为SD。

**图3:**
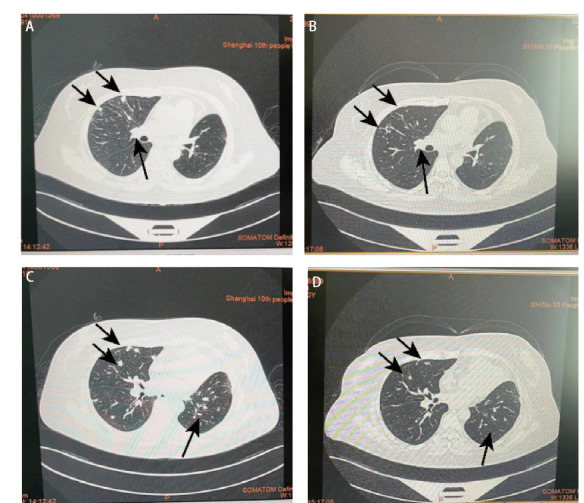
阿法替尼治疗前后对比。A、B：阿法替尼治疗前；C、D：阿法替尼治疗后（箭头所示病灶区域阿法替尼治疗后明显缩小）。

## 2 讨论

本文展现了阿法替尼联合安罗替尼在治疗EGFR L833V和H835L复合突变的NSCLC患者中的临床疗效。该患者具有如下特征：患者在确诊肺癌5年后出现异时性骨转移，5年间进行6次基因检测，直至2023年5月才检测出罕见基因复合突变；患者同时存在L833V和H835L两个罕见突变并且对阿法替尼联合安罗替尼治疗反应良好。

EGFR基因突变是NSCLC患者最常见的驱动基因，其中18-21号外显子的突变常见，尤其是19号外显子的缺失和21号外显子L858R点突变最为常见^[4]^。EGFR复合突变是酪氨酸激酶结构域中两个或多个独立突变的组合。复合突变的发生率具有很高的异质性，占全部EGFR突变患者的4%-26%^[6]^。复合EGFR突变的常见类型是EGFR经典突变和罕见突变的组合，两种或更多的罕见EGFR突变同时出现在NSCLC患者中极为罕见。但是随着NGS的不断发展和应用，越来越多的不常见EGFR突变被检测出来，如本案例患者EGFR L833V/H835L复合突变^[7,8]^。

EGFR-TKIs广泛应用于NSCLC患者的治疗当中，但是目前主要应用于EGFR常见突变的治疗中，并且展现出良好的疗效^[9]^。关于EGFR-TKIs对EGFR基因复合突变的治疗效果的研究得出的结论并不一致，部分研究^[4,9,10]^报道，EGFR复合突变对第二代EGFR-TKIs药物相较于EGFR单一突变更加敏感。Tu等^[11]^研究认为，EGFR基因复合突变患者与单一突变携带者相比，各项预后指标无明显差异。Liu等^[12]^研究认为，EGFR复合突变患者应用EGFR-TKIs治疗，预后比单一突变更差。2023年NSCLC中国临床肿瘤学会（Chinese Society of Clinical Oncology, CSCO）指南将第二代EGFR-TKIs药物如吉非替尼、阿法替尼等作为IV期驱动基因阳性NSCLC治疗方案的I级推荐。第二代EGFR-TKIs阿法替尼是ErbB家族抑制剂，阿法替尼可以通过不可逆地结合EGFR，抑制EGFR信号通路的激活，进而抑制癌细胞的生长、增殖和转移，并且结合人表皮生长因子受体2（human epidermal growth factor receptor 2, HER2）等位点可以进一步放大抑癌作用。目前三代EGFR-TKIs都已应用于肺癌的临床治疗，第一代EGFR-TKIs作用机制为可逆性结合EGFR，效果相对较弱；第二代EGFR-TKIs不可逆地结合EGFR，效果较好；第三代EGFR-TKIs则主要应用于存在T790M等耐药突变的情况，并且这类药更容易越过血脑屏障，在中枢神经系统中发挥作用^[5,13]^。虽然第二代EGFR-TKIs阿法替尼对T790M等耐药突变也有一定效果，但有效率不高。

EGFR L833V/H835L复合突变是一种十分罕见的复合突变形式，仅有少数报道（表1）。既往报道的11例患者均为EGFR L833V/H835L复合突变的肺腺癌患者，以亚洲人群为主，患者年龄最小为36岁，最大为89岁，且男性居多（男:女=7:4），容易发生转移。未发现吸烟与EGFR L833V/H835L复合突变之间存在的明显关系。Qin等^[14]^提示，EGFR T790M突变的出现可能是EGFR L833V/H835L复合突变的肺癌患者对第二代EGFR-TKIs获得性耐药的重要机制，并且在对阿法替尼耐药后，奥希替尼可以作为一种治疗选择。Yang等^[10]^报道患者因经济负担放弃第一推荐的阿法替尼而选择第二推荐的吉非替尼，吉非替尼展现出对EGFR L833V/H835L伴皮肤转移的NSCLC患者具有潜在的巨大疗效，该病例无进展生存期（progression-free survival, PFS）达18^+^个月。Long等^[4]^发现，以阿法替尼作为一线治疗药物治疗EGFR L833V/H835L复合突变NSCLC患者，疗效很好，PFS达10^+^个月。EGFR L833V突变常与H835L突变以复合突变的形式存在。L833V/H835L复合突变的NSCLC患者对第一代EGFR-TKIs治疗疗效往往不如经典突变的患者，但也存在少数患者比经典突变更加敏感，第二代EGFR-TKIs对EGFR L833V/H835L复合突变的NSCLC患者而言，效果更好。在本案例中，2023年5月患者基因检测结果发现EGFR L833V/H835L复合突变后，我们选择了阿法替尼进行治疗，并且表现出良好的临床疗效。

**表1:** EGFR L833V/H835L复合突变的NSCLC患者的临床特征及预后评价

Author	Gender	Age (yr)	Smoking status	Mutation type	Treatment	PFS (mon)
Long, et al.^[4]^	Male	65	Never smoked	L833V/H835L	Afatinib	>10
Yang, et al.^[10]^	Male	65	Current smoker (heavy smoker)	L833V/H835L	Gefitinib	>18
Qin, et al.^[14]^	Male	36	Never smoked	T790M/R670W/L833V/H835L	Afatinib，Axitinib	>7
Yang, et al.^[15]^	Male	89	Current smoker (light smoker)	L833V/H835L	Gefitinib	>8+
Zhuang, et al.^[16]^	Male	48	Never smoked	L833V/H835L	Pemetrexed+Cisplatin	>4
Frega, et al.^[17]^	Male	70	Past smoker	E709K/L833V/H835L	Afatinib	>2+
Li, et al.^[18]^	Female	77	Never smoked	L833V/H835L	Gefitinib	>15
Li, et al.^[19]^	Male	75	Never smoked	T790M/L833V/H835L	Afatinib，Axitinib	>13.5
Luo, et al.^[20]^	Female	53	Never smoked	L833V/H835L	Axitinib	>22
Smith, et al.^[21]^	Female	60	Past smoker	L833V/H835L	Axitinib	19
Yang, et al.^[22]^	Female	44	Never smoked	L833V/H835L	Ectinib	5

NSCLC: non-small cell lung cancer; PFS: progression-free survival.

本案例目前已使用阿法替尼联合安罗替尼治疗5个月余，患者临床症状明显减轻，病灶吸收良好，肿瘤相关指标明显下降，随访情况较好。临床上对于这类复合罕见基因突变的患者尚无确切的治疗方案，相关治疗药物的确切疗效及治疗机制仍不明确。但是鉴于复合罕见基因突变患者数量很少，难以设计前瞻性队列试验进行探究，因此单个的病例报告将有助于积累临床数据，为此类患者的治疗提供参考。

**Competing interests**

The authors declare that they have no competing interests.

## References

[1] Passaro A, Prelaj A, Bonanno L, et al. Activity of EGFR TKIs in Caucasian patients with NSCLC harboring potentially sensitive uncommon EGFR mutations. Clin Lung Cancer, 2019, 20(2): e186-e194. doi: 10.1016/j.cllc.2018.11.005 30563752

[2] Esteban E, Majem M, Martinez Aguillo M, et al. Prevalence of EGFR mutations in newly diagnosed locally advanced or metastatic non-small cell lung cancer Spanish patients and its association with histological subtypes and clinical features: The Spanish REASON study. Cancer Epidemiol, 2015, 39(3): 291-297. doi: 10.1016/j.canep.2015.02.003 25766256

[3] Shi Y, Au JS, Thongprasert S, et al. A prospective, molecular epidemiology study of EGFR mutations in Asian patients with advanced non-small-cell lung cancer of adenocarcinoma histology (PIONEER). J Thorac Oncol, 2014, 9(2): 154-162. doi: 10.1097/JTO.0000000000000033 24419411 PMC4132036

[4] Long X, Qin T, Lin J. Great efficacy of afatinib in a patient with lung adenocarcinoma harboring EGFR L833V/H835L mutations: a case report. Onco Targets Ther, 2020, 13: 10689-10692. doi: 10.2147/ott.S260157 33116645 PMC7585793

[5] Pang LL, Gan JD, Tan JR, et al. Efficacy and potential resistance mechanisms of afatinib in advanced non-small cell lung cancer patients with EGFR G719X/L861Q/S768I. Cancer, 2022, 128(21): 3804-3814. doi: 10.1002/cncr.34451 36069292

[6] Attili I, Passaro A, Pisapia P, et al. Uncommon EGFR compound mutations in non-small cell lung cancer (NSCLC): a systematic review of available evidence. Curr Oncol, 2022, 29(1): 255-266. doi: 10.3390/curroncol29010024 35049698 PMC8774526

[7] Nakamura H, Koizumi H, Sakai H, et al. Accuracy of the cobas EGFR mutation assay in non-small-cell lung cancer compared with three laboratory-developed tests. Clin Lung Cancer, 2018, 19(2): 170-174. doi: 10.1016/j.cllc.2017.10.015 29150249

[8] Xu J, Wu W, Wu C, et al. A large-scale, multicentered trial evaluating the sensitivity and specificity of digital PCR versus ARMS-PCR for detecting ctDNA-based EGFR p.T790M in non-small-cell lung cancer patients. Transl Lung Cancer Res, 2021, 10(10): 3888-3901. doi: 10.21037/tlcr-21-564 34858779 PMC8577974

[9] Sung H, Ferlay J, Siegel RL, et al. Global cancer statistics 2020: GLOBOCAN estimates of incidence and mortality worldwide for 36 cancers in 185 countries. CA Cancer J Clin, 2021, 71(3): 209-249. doi: 10.3322/caac.21660 33538338

[10] Yang X, Yao Y, Zhu Q. A L833V/H835L EGFR variant lung adenocarcinoma with skin metastasis: A case report and literature review. Heliyon, 2022, 8(12): e12080. doi: 10.1016/j.heliyon.2022.e12080 36531621 PMC9747578

[11] Tu HY, Ke EE, Yang JJ, et al. A comprehensive review of uncommon EGFR mutations in patients with non-small cell lung cancer. Lung Cancer, 2017, 114: 96-102. doi: 10.1016/j.lungcan.2017.11.005 29173773

[12] Liu HL, Han G, Peng M, et al. Efficacy of EGFR tyrosine kinase inhibitors in non-small cell lung cancer patients harboring different types of EGFR mutations: A retrospective analysis. J Huazhong Univ Sci Technolog Med Sci, 2017, 37(6): 864-872. doi: 10.1007/s11596-017-1819-4 29270745

[13] Liao BC, Lin CC, Yang JCH. Novel EGFR inhibitors in non-small cell lung cancer: current status of afatinib. Curr Oncol Rep, 2017, 19(1): 4. doi: 10.1007/s11912-017-0560-2 28138934

[14] Qin BD, Jiao XD, Yuan LY, et al. The effectiveness of afatinib and osimertinib in a Chinese patient with advanced lung adenocarcinoma harboring a rare triple EGFR mutation (R670W/H835L/L833V): a case report and literature review. Onco Targets Ther, 2018, 11: 4739-4745. doi: 10.2147/ott.S167346 30127622 PMC6091473

[15] Yang TY, Tsai CR, Chen KC, et al. Good response to gefitinib in a lung adenocarcinoma harboring a heterozygous complex mutation of L833V and H835L in epidermal growth factor receptor gene. J Clin Oncol, 2011, 29(16): e468-e469. doi: 10.1200/jco.2010.33.5802 21422421

[16] Zhuang Y, Xu J, Ma H, et al. A sequential method of epidermal growth factor receptor mutation detection reduces false negatives: a new case with doublet mutations of L833V and H835L in China. Clin Lung Cancer, 2013, 14(3): 295-300. doi: 10.1016/j.cllc.2012.11.003 23313172

[17] Frega S, Conte P, Fassan M, et al. A triple rare E709K and L833V/H835L EGFR mutation responsive to an irreversible pan-HER inhibitor: a case report of lung adenocarcinoma treated with afatinib. J Thorac Oncol, 2016, 11(5): e63-e64. doi: 10.1016/j.jtho.2016.01.023 27131295

[18] Li M, Zhou CZ, Yang JJ, et al. The in cis compound EGFR mutations in Chinese advanced non-small cell lung cancer patients. Cancer Biol Ther, 2019, 20(8): 1097-1104. doi: 10.1080/15384047.2019.1595280 30990107 PMC6605978

[19] Li T, Wang S, Ying J, et al. Afatinib treatment response in advanced lung adenocarcinomas harboring uncommon mutations. Thorac Cancer, 2021, 12(21): 2924-2932. doi: 10.1111/1759-7714.14156 34549528 PMC8563151

[20] Luo Z, Luo C, Zhou R, et al. Complete response to first-line osimertinib monotherapy in a complex epidermal growth factor receptor mutant (L833V/H835L ) lung adenocarcinoma patient: a case report. Anticancer Drugs, 2023, 34(8): 939-941. doi: 10.1097/cad.0000000000001523 37227041

[21] Smith JT, Puri S, Akerley W. Brief report: EGFR L833V/H835L duplex-mutated NSCLC with leptomeningeal carcinomatosis responsive to osimertinib. Clin Lung Cancer, 2023, 24(4): 360-361. doi: 10.1016/j.cllc.2023.01.010 36935243

[22] Yang Z, Jiang W, Zhang YW, et al. Icotinib combined with chemotherapy in the treatment of rare complex mutation of EGFR gene L833V and H835L in lung cancer:a case report. Zhongliu Yaoxue, 2023, 13(3): 385-388.

